# Correlation of Radial and Ulnar Shaft Bowing Using the 3D Central Line Method

**DOI:** 10.1002/jor.70288

**Published:** 2026-10-20

**Authors:** Christoph Zindel, Sylvano Mania, Tudor Trache, Michel Meisterhans, Julian Hasler, Lisa Reissner, Andreas Schweizer

**Affiliations:** ^1^ Department of Orthopedics Balgrist University Hospital University of Zurich Zurich Switzerland

**Keywords:** bowing, central line method, forearm, radius, ulna

## Abstract

Forearm rotation depends on the multiplanar bowing of the radius and ulna. Despite the importance of the fundamental 3D anatomic understanding of the forearm for fracture fixation or implant design, all morphological studies have applied only two‐dimensional methods rather than analyzing the true full 3D space. No study investigated the intra‐pair relation of ulna/radius either. Therefore, we processed CT scans of 30 healthy forearms into 3D models. Each bone was divided into 10 segments along the rotation axis, creating 11 cross‐sections with central points. Connecting these central points yielded the Central‐Line. Bowing was quantified as the Bowing‐Index (area under the Central‐Line). Bowing was analyzed in ap, sagittal and the newly defined Main‐Deformity‐Axis (MDA) plane. Maximal radial deviation averaged 6.4 ± 2.3 mm in frontal, 1.3 ± 3.3 mm sagittal, and 7.2 ± 1.8 mm in MDA plane over all forearms. Maximal ulnar deviation was greater with 8.2 ± 2.9 mm frontal, 9.5 ± 1.7 mm sagittal, and 12.3 ± 2.1 mm in MDA. MDA orientation angle (angular tilt in the dorso‐radial direction in the axial plane) was dorso‐radial, with significantly larger out‐of‐plane bowing in the ulna (60.4° vs. 15.0°; *p* < 0.01), yielding an average intra‐bone‐pair‐difference of 45.5° ± 33.0°. A negative correlation was found between radius and ulna MDA angles (*r* = −0.40, *p* = 0.030). The Bowing Index was higher in the ulna across all planes (*p* ≤ 0.011), with a significant negative correlation between Ulna and Radius (MDA plane, *r* = −0.38, *p* = 0.036). Distal ulna analysis revealed consistent dorsal and slight ulnar tilt. The Central Line Method enables 3D characterization of forearm bowing, identifying deformity planes beyond frontal/sagittal axes. For the first time, significant negative correlations between radial and ulnar bowing were demonstrated. These insights hold clinical relevance for fracture fixation, osteotomy planning, and implant design.

## 1 Introduction

The forearm enables pronation and supination of the upper limb through the coordinated interaction of the radius and ulna. This rotational function depends on the unique multiplanar geometry of both bones—the bowing. Because of their mechanical interdependence, deformities in either bone can substantially affect motion and overall limb function [1, 2, 3, 4, 5, 6]. The radius exhibits a characteristic bowing in the frontal (ap) plane, and restoring this bow in terms of both magnitude and location has been shown to correlate with improved outcomes following fracture fixation [4, 7]. Similarly, malalignment of the proximal ulnar bowing after injury can result in compromised rotational mechanics, subluxation, early joint degeneration, and functional deficits [8, 9, 10]. While most studies on radial bowing have initially focused on the frontal (ap) plane, now more attention has been given as well to its sagittal (lateral) contour [11]. In contrast, investigations of ulnar bowing have predominantly addressed proximal segments, particularly in relation to implant design for fracture fixation and elbow arthroplasty [12, 13, 14], and only few studies analyzed the whole ulna [11]. To date, no study has specifically investigated the bowing morphology of the distal ulna.

Most morphological measurement techniques assessing radial and/or ulnar bowing have historically relied on two‐dimensional radiographs [4, 5, 6, 7, 15, 16, 17] or digital photographs of cadaveric bones [11]. These methods typically focused on quantifying the maximum deviation and its corresponding location [4, 5, 7, 11, 15, 16, 17], and measurements were exclusively performed in strict frontal (ap) and sagittal (lateral) planes. While more recent studies have employed computed tomography (CT) for evaluating bone curvature [18, 19, 20], they have continued to apply conventional two‐dimensional parameters and methods—namely maximum deviation and its location—without extending the analyses into the full 3D space. Some studies have compared morphological findings to the contralateral limb [11, 18]; however, to date, no study has investigated potential correlations between radial and ulnar bowing within the same forearm pair.

Therefore, the aim of the present study was [1] to introduce a novel Central Line Method for quantifying three‐dimensional bone morphology and [2] to explore previously unrecognized spatial correlations between the radius and ulna that may only be detectable in 3D space. Additionally [3], a focused analysis of the distal ulna was conducted to better characterize its geometric properties. We hypothesized that [1] the Central Line Method is a reliable approach for detecting geometrical properties, particularly bowing [2], a correlation exists between radial and ulnar bowing magnitude, and [3] the distal ulna demonstrates not only an offset but also a consistent slope relative to the forearm axis.

## 2 Materials and Methods

The study was approved by the local ethical committee (Zurich Cantonal Ethics Commission, BASEC‐Nr. Req‐2021–00223) and all patients gave their informed consent. This is a Level III Diagnostic Study.

This study was performed on computed tomography data (CT, slice thickness 1 mm; 120 kV; Philips Brilliance 40 CT, Philips Healthcare, The Netherlands) from healthy forearms of 30 patients. The selected patients had undergone these scans for contralateral forearm deformities, allowing the nonaffected side to serve as a healthy template for 3D‐guided patient‐specific osteotomies [21, 22]. Commercial segmentation software, Mimics (Materialise, Leuven, Belgium), was utilized to segment the CT data and generate 3D surface models of the forearm [23, 24, 25]. These models were then imported into our in‐house developed CAD software for preoperative planning (CASPA, Balgrist CARD AG, Zurich, Switzerland) as described in literature before [26, 27, 28, 29, 30, 31]. All left forearms are mirrored to be comparable to the right forearm models.

First, the functional forearm rotation axis (FFRA) was determined for each bone model. As described earlier [3, 32], the movement of the radius around the ulna can be explained using a single rotation axis, without involving the humerus as a reference point. This can be simplified as a line extending from the center of the radial head to the ulnar fovea. This line is considered as FFRA. The adjustment of the rotation axis on the ulnar head was manually carried out, with the goal of consistently preserving a constant distance between the sigmoid notch and the articular surface of the ulnar head throughout the entire range of motion (ROM). Additionally, efforts were made to maintain an appropriate sphericity of the radial head. All bone models were aligned as a true supinated position at 90°.

### 2.1 Central Line Method and Coordinate System

The radius and ulna were divided along the FFRA into 10 equal parts to obtain 11 radial and ulnar cross‐sections. The central point (CP) of each cross‐section was calculated as the centroid of its cross‐sectional area. The connection of these central points led to the central line of each radius and ulna consisting of 11 central points (CP) and 10 central line segments (CLS) each. The central line was then analyzed in regard of bowing in the 3D space. An example is illustrated in Figure 1.

**FIGURE 1:**
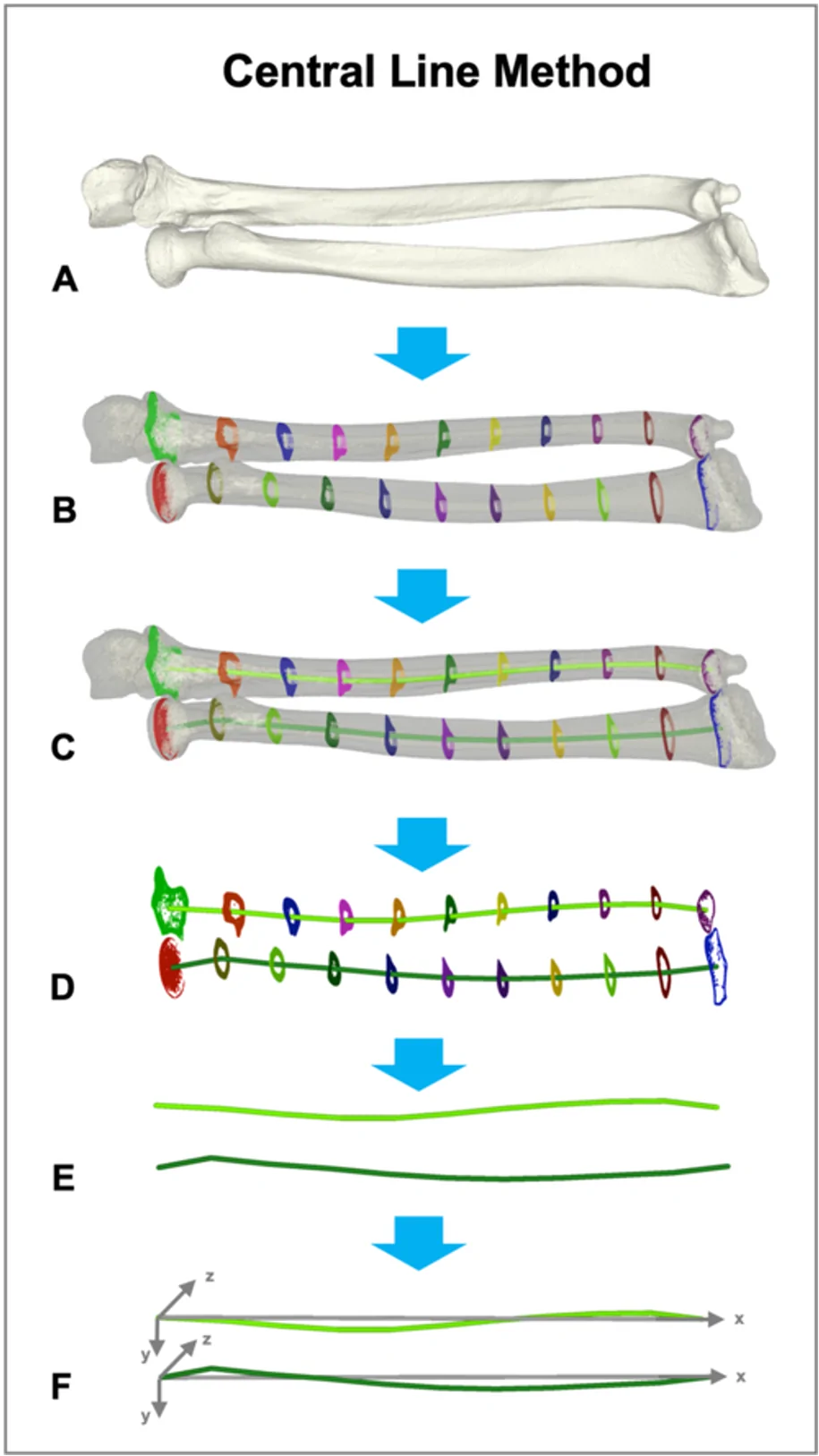
Schematic illustration of the workflow for analyzing 3D bone bowing using the central‐line method, shown in the frontal (radio‐ulnar) plane (A) 3D bone model of the ulna and radius. (B) Dividing the radius and ulna into 10 equal parts to obtain 11 radial and ulnar cross‐sections with a central point each. (C) Connecting the central points leads to a central line of each bone (ulna: light green, radius: dark green). (D–F) Subtracting the central line for the analyses of the 3D bowing.

An individual coordinate system was defined for each radius and ulna separately (see an example in Figure 1F). The *x*‐axis represents the connecting line between the central point of the most proximal and the most distal cross‐section of the radius, respectively the ulna. The *y*‐axis represents the radio‐ulnar axis pointing toward radially. It is parallel to the humero‐ulnar joint, which is simulated as a line within a cylinder that fits the ulnar trochlea joint. The consequential *z*‐axis points toward dorsal and represents the dorso‐palmar axis. The planes are defined as frontal plane (radio‐ulnar plane) normal to the *z*‐axis, sagittal plane (dorso‐palmar plane) normal to the *y*‐axis and axial plane normal to the *x*‐axis.

### 2.2 Definition of Deviation, Slope, Bowing, Main Deformity Axis, and Bowing Index

Based on the central line—comprising 11 central points (CP) and 10 central line segments (CLS)—the remaining parameters were derived and calculated. First, for each CP, the deviation in the two principal anatomical planes was analyzed: the *y*‐axis representing radial deviation (mm) and the *z*‐axis representing dorsal deviation (mm).

These deviations were used to compute the orientation of the Main Deformity Axis (MDA, in degrees (°)), defined as the angular tilt in the dorso‐radial direction in the axial plane, and was calculated trigonometrically from the ratio of the total cumulative deviation in the *y*‐ and *z*‐directions. The MDA plane was defined as plane spanned by the MDA axis and the FFRA.

The slope—defined as the angular orientation of a CLS relative to the respective axis—was calculated for each segment in the *y*‐plane (radial slope, in degrees (°)), *z*‐plane (dorsal slope, in degrees (°)), and MDA direction (MDA slope, in degrees (°)).

Bowing was defined as the angular deviation between two consecutive CLSs. It was computed analogously to the slope in the *y*‐direction (radial bowing, in degrees (°)), *z*‐direction (dorsal bowing, in degrees (°)), and along the MDA (MDA bowing, in degrees (°)).

If the central line is considered as a mathematical function *f*(*x*) in one plane, then the slope corresponds to its first derivative *f*′(*x*), and the bowing corresponds to its second derivative *f*″(*x*) in that plane.

From these data, the absolute maximal deviation (mm), the maximum slope (°), and the maximum bowing (°) per bone in the two principal directions and along the MDA were analyzed. Additionally, the location of the maximal deviation, maximal slope, and maximal bowing was recorded as a percentage of total bone length from proximal to distal.

Subsequently, correlation analyses were performed between paired radius and ulna to evaluate associations between the magnitude and location of the respective maxima.

The direction of the MDA was also assessed for all radii and ulnae, and potential correlations within the paired radius–ulna bones were evaluated. The Bowing Index was defined as the normalized area under the curve (mathematically the integration of *f*(*x*) in one plane) and analyzed in the two principal plane and the MDA plane. Correlation analyses for the Bowing Index were again performed within corresponding radius–ulna pairs. The Bowing Index represents the average bowing over the entire length of the bone, rather than being based on a single measurement point such as the maximum deviation. It is therefore considered the most appropriate metric for comparing overall bowing between different bones.

### 2.3 Statistical Analysis

All measurements and calculated parameters—including deviation, slope, bowing, MDA, and Bowing Index, as well as their respective maxima and positional locations—were reported as mean, standard deviation, and range. The location along the *x*‐axis was normalized, with 0% representing the most proximal point and 100% the most distal point. The directional definitions for all deviation, slope, and bowing parameters were standardized as follows: radial deviation and radial slope were defined as positive when directed toward the radial side, and negative when directed ulnarly. Dorsal deviation and dorsal slope were defined as positive when directed dorsally and negative when directed palmarly. The MDA was defined as a positive angular tilt in the dorso‐radial direction and as negative in the ulno‐palmar direction, measured from the frontal plane. Deviations and slopes along the MDA were considered positive when directed toward the MDA direction and negative when oriented in the opposite direction (180° opposite to the MDA). Radial bowing was defined as positive when the apex pointed toward the radial side and negative when the apex was directed ulnarly. Dorsal bowing was considered positive when the apex pointed dorsally and negative when oriented palmarly. Bowing along the MDA was considered positive when the apex pointed in the direction of the MDA and negative when pointing in the opposite direction. From a mathematical perspective, this corresponds to the sign of the second derivative *f*″(*x*), with positive bowing representing positive curvature of the function *f*(*x*) describing the central line. The Bowing Index, being an area‐based parameter, was inherently positive by definition.

The normality of all variables was assessed using the Shapiro–Wilk test. Depending on data distribution, either a two‐tailed unpaired *t*‐test or the Mann–Whitney U test was applied for group comparisons. Pearson correlation analysis was used to evaluate the relationships between corresponding parameters. Owing to the highly standardized and predominantly automated measurement procedure, no inter‐ or intra‐reader reliability analysis was performed [3].

A *p*‐value of less than 0.05 was considered statistically significant. All statistical analyses were performed using SPSS Statistics for Windows (version 26.0; IBM).

## 3 Results

The ulna demonstrates significantly higher maximal axis deviation compared to the radius in the frontal (ulna: 8.2 ± 2.9 mm; radius: 6.4 ± 2.3 mm, *p* < 0.01), sagittal (ulna: 9.5 ± 1.7 mm; radius: 1.3 ± 3.3 mm, *p* < 0.01), and MDA plane (ulna: 12.3 ± 2.1 mm; radius: 7.2 ± 1.8 mm, *p* < 0.01). The location of maximal axis deviation was significantly more proximal in the ulna compared to the radius in all planes (frontal: 27.3% ± 9.7% vs. 52.7% ± 8.6%, *p* < 0.01; sagittal: 26.7% ± 8.8% vs. 38.8% ± 18.5%, *p* < 0.01; MDA: 25.2% ± 5.6% vs. 53.0% ± 3.4%, *p* < 0.01). The location of the maximal axis deviation is marked in Figure 2 as Asterix (ulna: light green, radius: dark green) for all planes. No significant correlation was found between the ulna and radius regarding either the magnitude or the location of the maximal deviation.

**FIGURE 2:**
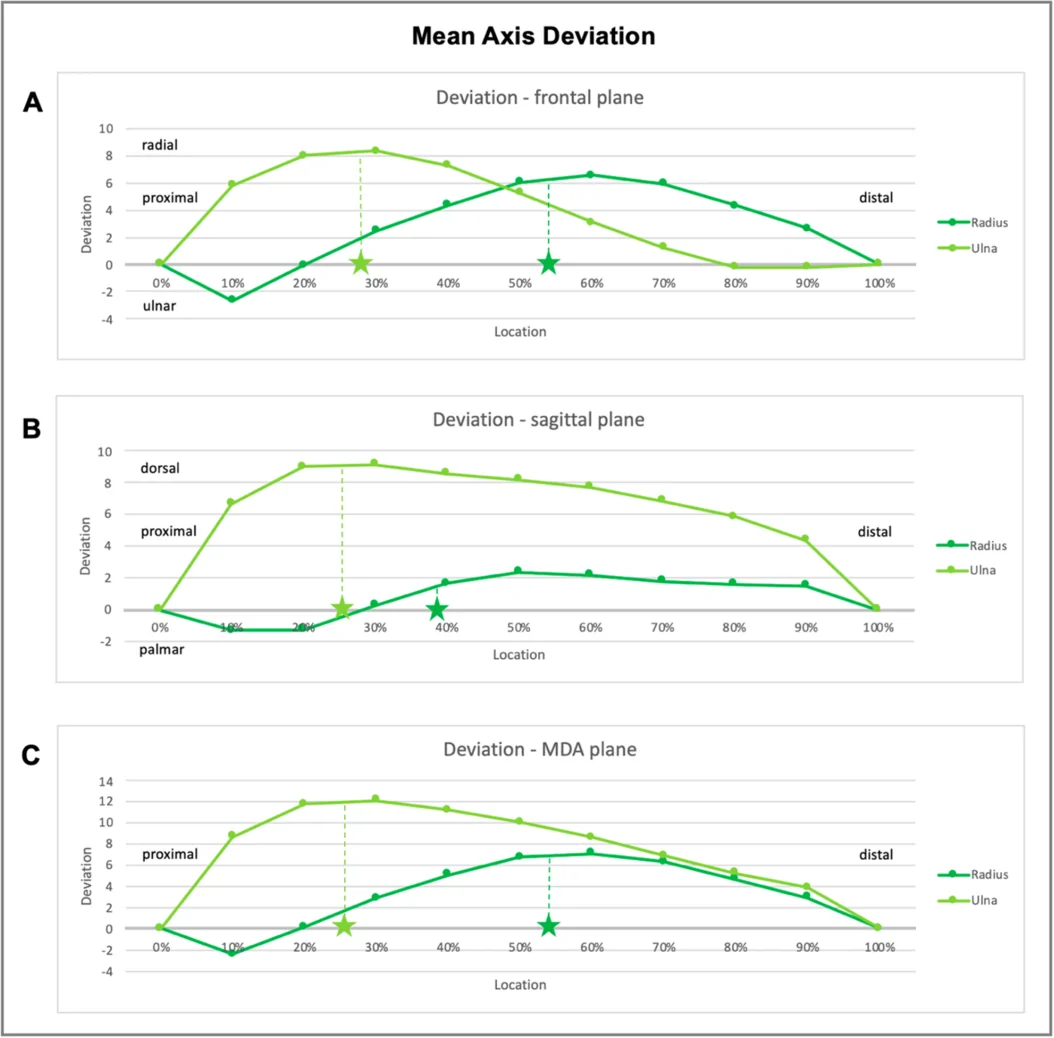
Mean axis deviation in the (A) frontal, (B) sagittal, and (C) MDA plane for the ulna (light green) und radius (dark green) with the location of 0% being proximal and of 100% being distal. The asterisk indicates the location of the mean maximum.

The maximum bowing in the frontal plane was significantly higher in the radius than in the ulna (ulna: −8.2° ± 4.8°; radius: 12.8° ± 5.1°, *p* < 0.01). The ulna showed significantly greater maximal bowing in the sagittal (ulna: −11.4° ± 3.1°; radius: 3.7° ± 6.0°, *p* < 0.01) and MDA plane (ulna: 13.4° ± 3.4°; radius: 4.1° ± 1.1°, *p* < 0.01). The location of maximum bowing was significantly more proximal in the MDA plane in the ulna (ulna: 0.7% ± 2.8%; radius: 33.3% ± 18.4%, *p* < 0.01) but not significant in the frontal (ulna: 8.1% ± 20.1%; radius: 3.0% ± 16.0%) and sagittal plane (ulna: 19.3% ± 35.0%; radius: 25.9% ± 35.4%). No significant correlation was found between the ulna and radius regarding either the magnitude or the location of the maximal bowing. A comprehensive summary of all maximum values and their respective locations is provided in Table 1, while additional data on deviation, slope, and bowing can be found in Appendix 1.

**TABLE 1:** Maximum and location of maximum. All values are reported as mean (SD) in either mm, degrees (°), or % as marked. MDA, Main Deformity Axis.

Maximum and location of maximum				
	Radius	Ulna	*T*‐test	Pearson ‐ correlation	
	(*n* = 30)	(*n* = 30)	*p*‐value	*r*‐value	*p*‐value
Deviation	[mm]	[mm]			
Radial deviation					
Maximum	6.4 (2.3)	8.2 (2.9)	< 0.01*	0.13	0.478
Location	52.7% (8.6%)	27.3% (9.7%)	< 0.01*	0.00	1.000
Dorsal deviation					
Maximum	1.3 (3.3)	9.5 (1.7)	< 0.01*	−0.16	0.400
Location	38.8% (18.5%)	26.7% (8.8%)	< 0.01*	−0.30	0.111
MDA deviation					
Maximum	7.2 (1.8)	12.3 (2.1)	< 0.01*	−0.03	0.888
Location	53.0% (3.4%)	25.2% (5.6%)	< 0.01*	0.12	0.524
Slope	[°]	[°]			
Radial slope					
Maximum	3.5 (8.7)	−14.0 (2.9)	< 0.01*	0.10	0.680
Location	34.3% (39.2%)	5.0% (5.0%)	< 0.01*	0.00	1.000
Dorsal slope					
Maximum	3.2 (5.4)	−12.6 (10.9)	< 0.01*	0.02	0.914
Location	43.0% (39.4%)	12.0% (30.6%)	< 0.01*	0.32	0.086
MDA slope					
Maximum	9.7 (2.0)	21.4 (3.4)	< 0.01*	0.00	0.984
Location	41.0% (39.4%)	5.0% (5.0%)	< 0.01*	0.00	1.000
Bowing	[°]	[°]			
Radial bowing [°]					
Maximum	12.8 (5.1)	−8.2 (4.8)	< 0.01*	0.09	0.654
Location	3.0% (16.0%)	8.1% (20.1%)	0.140	−0.08	0.692
Dorsal bowing [°]					
Maximum	3.7 (6.0)	−11.4 (3.1)	< 0.01*	0.02	0.922
Location	25.9% (35.4%)	19.3% (35.0%)	0.237	0.35	0.057
MDA bowing [°]					
Maximum	4.1 (1.1)	13.4 (3.4)	< 0.01*	−0.17	0.370
Location	33.3% (18.4%)	0.7% (2.8%)	< 0.01*	0.00	1.000

*Note:* Data are presented as mean (SD).

* *p* < 0.05.

The direction of the MDA was for ulna and radius mainly dorso‐radial orientated and the mean direction of the MDA per forearm was 37.7° ± 12.0°. The direction of the MDA was significantly more pronounced in the ulna (60.4° ± 12.4°) compared to the radius (15.0° ± 26.1°, *p* < 0.01). The average difference was 45.5° ± 33.0°. A significant moderate negative correlation was observed between radius and ulna MDA direction (*r* = −0.40, *p* = 0.030). The direction of the MDA is illustrated in Figure 3 as dashed arrow (ulna: light green, radius: dark green).

**FIGURE 3:**
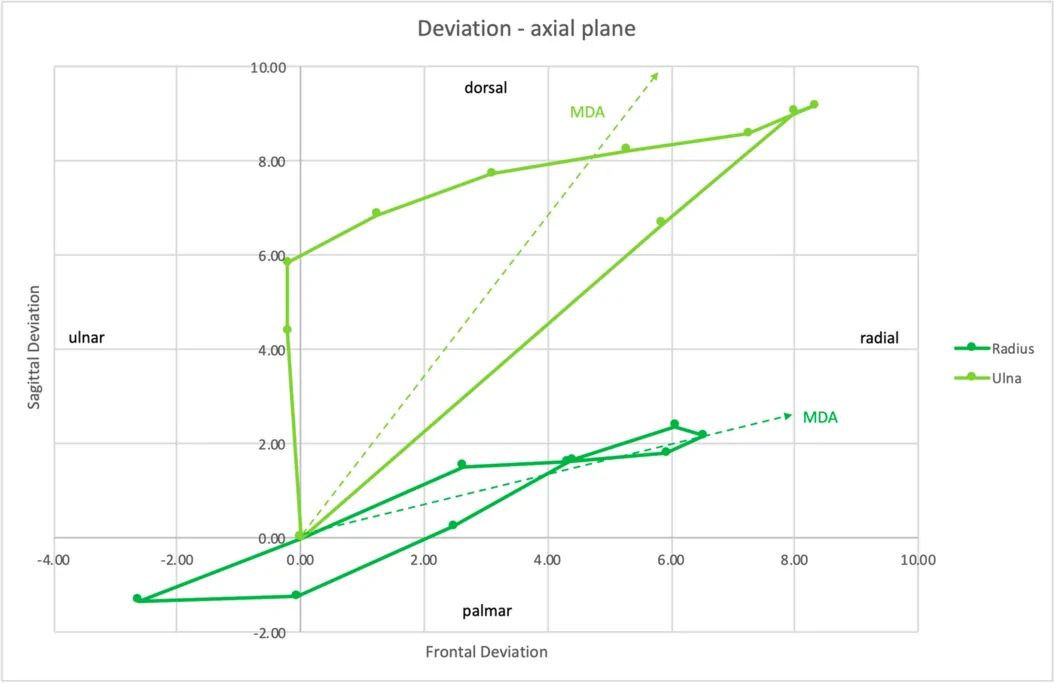
Mean axis deviation in the axial plane for the ulna (light green) and radius (dark green). The arrow illustrates the orientation of the average Main Deformity Axis (MDA).

The Bowing Index was significantly higher in the ulna in all three planes (frontal: 3.9 ± 1.7 vs. 3.0 ± 1.2 (*p* = 0.011), sagittal: 6.6 ± 1.3 vs. 0.9 ± 1.3 (*p* < 0.01), MDA: 8.6 ± 1.5 vs. 3.4 ± 1.1 (*p* < 0.01)). A significant negative correlation was found for the Bowing Index in the MDA plane (*r* = −0.38, *p* = 0.036). Table 2 provides detailed information on MDA and Bowing Index.

**TABLE 2:** Main Deformity Axis and Bowing Index. All values are reported as mean (SD). MDA, Main Deformity Axis.

Main Deformity Axis and Bowing Index					
	Radius	Ulna	*T*‐test	Pearson ‐ correlation	
	(*n* = 30)	(*n* = 30)	*p*‐value	*r*‐value	*p*‐value
Main Deformity Axis					
Radio‐dorsal tilt [°]	15.0 (26.1)	60.4 (12.4)	< 0.01*	−0.40	0.030*
Bowing Index					
Frontal plane	3.0 (1.2)	3.9 (1.7)	0.011*	0.00	0.994
Sagittal plane	0.9 (1.3)	6.6 (1.3)	< 0.01*	−0.18	0.333
MDA plane	3.4 (1.1)	8.6 (1.5)	< 0.01*	−0.38	0.036*

*Note:* Data are presented as mean (SD).

* *p* < 0.05.

Additional analysis of the distal ulna revealed that, within the distal 22 mm of the bone length, the bone is tilted 0.6° ± 3.6° ulnarly and 11.1° ± 3.0° dorsally. When considering the first 44 mm, the tilt measures 0.3° ± 2.6° ulnarly and 7.6° ± 1.8° dorsally. Table 3 provides more detailed information on the analysis of the distal ulna across all anatomical planes.

**TABLE 3:** Analysis of the distal ulna across all planes for the first 22 mm and for the first 44 mm. All values are reported as mean (SD) in either mm, degrees (°), or % as marked. MDA, Main Deformity Axis.

	First 22 mm	First 44 mm	*T*‐test	Pearson ‐ correlation	
Analysis distal ulna	(*n* = 30)	(*n* = 30)	*p*‐value	*r*‐value	*p*‐value
Deviation	[mm]	[mm]			
Radial deviation	−0.2 (1.5)	−0.2 (2.1)	0.494	0.94	< 0.01*
Dorsal deviation	4.4 (1.2)	5.8 (1.4)	< 0.01*	0.91	< 0.01*
MDA deviation	3.9 (1.0)	5.3 (1.3)	< 0.01*	0.90	< 0.01*
Slope	[°]	[°]			
Radial slope	−0.6 (3.6)	−0.3 (2.6)	0.362	0.94	< 0.01*
Dorsal slope	11.1 (3.0)	7.6 (1.8)	< 0.01*	0.92	< 0.01*
MDA slope	−10.0 (2.7)	−6.8 (1.7)	< 0.01*	0.90	< 0.01*
Bowing	[°]	[°]			
Radial bowing	0.6 (3.1)	3.7 (1.6)	< 0.01*	0.23	0.219
Dorsal bowing	−7.4 (3.1)	−1.1 (1.2)	< 0.01*	−0.03	0.878
MDA bowing	6.4 (2.7)	−0.7 (1.5)	< 0.01*	−0.04	0.836

*Note:* Data are presented as mean (SD).

* *p* < 0.05.

## 4 Discussion

To the best of our knowledge, this is the first study to investigate forearm bowing outside the conventional frontal and sagittal planes, and to analyze correlations in bowing between the radius and ulna within the same forearm pair.

The major finding is a significant negative correlation in bowing along the Main Deformity Axis (MDA), indicating that increased curvature of one bone is associated with decreased curvature of the other. This observation is biomechanically plausible, given that during pronation and supination, the radius rotates around the relatively stationary ulna [5, 7], but could not yet be proven due to the lack of three‐dimensional analyses methods in literature.

Second, the three‐dimensional analysis revealed novel principal deformity axes of bowing that lie outside the conventional radiological planes (i.e., frontal and sagittal). For the radius, the main deformity axis (MDA) was oriented predominantly in the radial direction with a lesser dorsal component, whereas for the ulna, the MDA was directed more dorsally with a lesser radial inclination. The mean angular difference between the MDAs of the radius and ulna was 45.5° ± 33.0°, with a statistically significant negative correlation. This indicates that the steeper the MDA of one bone, the flatter it tends to be in the corresponding contralateral bone. To our knowledge, this interdependent angular relationship between radius and ulna bowing orientation has not been previously described in literature.

Furthermore, the results from our quasi two‐dimensional analyses of the location of maximal deviation in the classic planes (frontal and sagittal) align closely with previously in literature reported values. For example, Weber et al. [11] reported the location of maximal radial bowing in the frontal plane at 58.2% ± 4.4% of the bone length (our study: 52.7% ± 8.6%), and in the sagittal plane at 45% ± 11% (our study: 38.8% ± 18.5%). For the ulna, they observed maximal deviation in the frontal plane at 31.7% ± 3.6% (our study: 27.3% ± 9.7%) and in the sagittal plane at 33% ± 15% (our study: 26.7% ± 8.8%). Hreha et al. [33] found a frontal plane bowing location in the radius at 59% ± 5% (our study: 52.7% ± 8.6%) and in the ulna at 75% ± 8% (our study: 27.3% ± 9.7%), as well as in the sagittal plane of the ulna at 39% ± 13% (our study: 26.7% ± 8.8%). The substantial discrepancy in the frontal plane measurements for the ulna can be attributed to the measurement technique used by Hreha et al., where the bowing span was defined from the most radial point of the proximal ulna to the most radial point of the ulnar styloid. This approach omits the proximal portion of the ulnar curvature, thereby shifting the apparent bowing location more distally compared to our measurements. Hong et al. [18] reported the frontal plane radial bowing location at 56% (our study: 52.7% ± 8.6%), sagittal plane at 64% (our study: 38.8% ± 18.5%), and for the ulna at 35% in the frontal plane (our study: 27.3% ± 9.7%) and 25% in the sagittal plane (our study: 26.7% ± 8.8%). The deviation in sagittal radial bowing values is likely due to methodological differences; Hong et al. defined the distal limit of their tangent based on the most volar cortical point at the bicipital tubercle, placing the reference line far from the bone, which systematically shifts the point of maximal deviation more distally. Shuman et al. [20] analyzed ulnar bowing in frontal plane only, reporting a maximal deviation at 36.7% ± 3.2% (our study: 27.3% ± 9.7%). Kass et al. [17] examined solely radial bowing in children and adolescents (ages 0–17 years) in frontal plane and found a consistent deviation at 61.7% ± 4.5% (our study: 52.7% ± 8.6%) over the evolving age.

It should be noted that the magnitude of maximal deviation reported in our study is not directly comparable with values in existing literature. Traditional methods [4, 7] measure bowing as a deviation from a surface‐based tangent, whereas the novel “reduce‐to‐line” method used in our study quantifies the deviation of the central axis.

Additionally, our study is the first to analyze the distal ulna in 3D with respect to bowing and tilt. We identified a consistent angular tilt of the distal ulna, measured as 0.6° ± 3.6° ulnarly and 11.1° ± 3.0° dorsally within the first 22 mm, and 0.3° ± 2.6° ulnarly and 7.6° ± 1.8° dorsally within the first 44 mm. These findings may help to explain the challenges associated with traditional ulnar head prostheses—specifically limited range of motion and joint instability—as previously reported by Furrer et al. [22]. Beyond the frequently undersized offset and its suboptimal orientation described by Furrer et al., neglecting the additional angular tilt identified in our study may further contribute to these complications. Consequently, future implant designs or surgical techniques involving ulnar head prostheses should consider this angulation during positioning and implantation.

### 4.1 Limitations

A limitation of the current study is the limited sample size, although it is important to acknowledge that the sample size is comparable to other three‐dimensional analyses with similar methods [3, 34, 35]. Second, incorporating additional clinical scores would further complement the study. However, as our primary focus was on morphology, the clinical score would not add further information to answer our hypothesis. In addition, the present study is subject to the common limitations of computer‐assisted 3D analyses, including potential segmentation errors and dependence on the selected coordinate system. As nearly all methodological steps were fully automated, we deliberately did not perform an intra‐ or interobserver reliability analysis, as the influence of user‐dependent variability was considered minimal.

## 5 Conclusions

The newly introduced central line method is a groundbreaking method to describe bowing of forearm bones enabling to find relevant deformity planes out of the standard frontal and sagittal planes. For the first time in literature, a statistically significant negative correlation was demonstrated between radial and ulnar bowing, indicating that increased curvature in one bone is associated with reduced curvature in the other. Additionally, the angular orientation of the MDA was found to correlate negatively between the radius and ulna. These findings provide clinically relevant morphological insights with potentially direct implications for fracture fixation, corrective osteotomy, and the design of orthopedic implants, such as ulnar head prostheses.

## Author Contributions

**Christoph Zindel:** conceptualization, data acquisition, formal analysis, investigation, methodology, project administration, software, validation, visualization, writing – original draft. **Sylvano Mania:** conceptualization, data acquisition, methodology, formal analysis, writing – review and editing. **Tudor Trache:** data acquisition, formal analysis. **Michel Meisterhans:** conceptualization, data acquisition, formal analysis. **Julian Hasler:** data acquisition, formal analysis. **Lisa Reissner:** conceptualization, formal analysis supervision, writing – review and editing. **Andreas Schweizer:** conceptualization, formal analysis, investigation, methodology, supervision, validation, writing – review and editing. All authors have read and approved the final submitted manuscript.

## Supporting information


Supporting File

## Data Availability

The data that support the findings of this study are available from the corresponding author upon reasonable request.
