# Supplementary material for: Correlation of Radial and Ulnar Shaft Bowing Using the 3D Central Line Method
Source: J Orthop Res. 2026 Oct 20;44(10):e70288. doi: 10.1002/jor.70288 (PMC13684049; doi:10.1002/jor.70288)
Supplement: Supplementary file 1 [file JOR-44-0-s001.docx]

**Deviation, Slope and Bowing**

| | Radius (n = 30) | Ulna (n = 30) | $p$-Value | | Radius (n = 30) | Ulna (n = 30) | $p$-Value | | Radius (n = 30) | Ulna (n = 30) | $p$-Value |
|---|---|---|---|---|---|---|---|---|---|---|---|
| **Deviation** | [mm] | [mm] | | **Slope** | [mm] | [mm] | | **Bowing** | [mm] | [mm] | |
| Radial Deviation | | | 0.283 | Radial Slope | | | 0.489 | Radial Bowing | | | 0.244 |
| CP 1 | 0.0 (0.0) | 0.0 (0.0) | | CLS 1 | 6.8 (3.1) | -14 (2.9) | | CLS 1-2 | 13.2 (3.7) | -8.6 (3.3) | |
| CP 2 | -2.6 (1.3) | 5.8 (1.3) | | CLS 2 | -6.4 (2.0) | -5.4 (2.0) | | CLS 2-3 | 0.0 (3.1) | -4.6 (1.7) | |
| CP 3 | -0.1 (1.5) | 8.0 (1.7) | | CLS 3 | -6.4 (1.7) | -0.8 (1.7) | | CLS 3-4 | -1.6 (1.6) | -3.6 (1.7) | |
| CP 4 | 2.5 (1.4) | 8.3 (2.1) | | CLS 4 | -4.9 (1.4) | 2.7 (1.5) | | CLS 4-5 | -0.6 (1.3) | -2.3 (1.4) | |
| CP 5 | 4.4 (1.6) | 7.3 (2.3) | | CLS 5 | -4.3 (1.1) | 5.0 (1.1) | | CLS 5-6 | -3.1 (1.2) | -0.6 (1.2) | |
| CP 6 | 6.1 (1.7) | 5.3 (2.4) | | CLS 6 | -1.2 (1.2) | 5.6 (1.3) | | CLS 6-7 | -2.8 (1.5) | 0.8 (1.4) | |
| CP 7 | 6.5 (1.8) | 3.1 (2.5) | | CLS 7 | 1.6 (1.0) | 4.8 (1.2) | | CLS 7-8 | -2.5 (1.4) | 1.1 (1.2) | |
| CP 8 | 5.9 (1.6) | 1.2 (2.3) | | CLS 8 | 4.1 (1.3) | 3.8 (1.6) | | CLS 8-9 | -0.3 (1.1) | 3.7 (1.6) | |
| CP 9 | 4.3 (1.2) | -0.2 (2.1) | | CLS 9 | 4.4 (1.5) | 0.1 (2.1) | | CLS 9-10 | -2.4 (2.7) | 0.6 (3.1) | |
| CP 10 | 2.6 (1.0) | -0.2 (1.5) | | CLS 10 | 6.8 (2.6) | -0.6 (3.6) | | ***AVG*** | ***0.0 (4.8)*** | ***1.5 (3.5)*** | |
| CP 11 | 0.0 (0.0) | 0.0 (0.0) | | ***AVG*** | ***0.0 (5.1)*** | ***-0.1 (5.7)*** | | | | | |
| ***AVG*** | ***2.7 (2.9)*** | ***3.5 (3.4)*** | | | | | | | | | |
| Dorsal Deviaton | | | < 0.01* | Dorsal Slope | | | 0.489 | Dorsal Bowing | | | 0.034* |
| CP 1 | 0.0 (0.0) | 0.0 (0.0) | | CLS 1 | 3.4 (2.9) | -16.1 (3.5) | | CLS 1-2 | 3.7 (4.4) | -10.2 (4.1) | |
| CP 2 | -1.3 (1.2) | 6.7 (1.5) | | CLS 2 | -0.3 (2.5) | -6.1 (2.0) | | CLS 2-3 | 3.5 (2.4) | -5.7 (1.8) | |
| CP 3 | -1.2 (1.3) | 9.0 (1.7) | | CLS 3 | -3.8 (2.1) | -0.4 (1.7) | | CLS 3-4 | -0.2 (1.7) | -2.0 (1.8) | |
| CP 4 | 0.3 (1.7) | 9.2 (1.8) | | CLS 4 | -3.6 (1.4) | 1.6 (1.4) | | CLS 4-5 | -1.8 (1.3) | 0.6 (2.1) | |
| CP 5 | 1.7 (2.0) | 8.6 (1.8) | | CLS 5 | -1.8 (1.0) | 1.0 (2.1) | | CLS 5-6 | -2.3 (1.3) | -0.3 (2.3) | |
| CP 6 | 2.4 (2.2) | 8.2 (2.1) | | CLS 6 | 0.5 (1.1) | 1.3 (1.3) | | CLS 6-7 | -0.5 (1.2) | -1.0 (1.0) | |
| CP 7 | 2.2 (2.1) | 7.7 (1.9) | | CLS 7 | 0.9 (1.4) | 2.2 (1.4) | | CLS 7-8 | 0.5 (0.7) | -0.3 (1.3) | |
| CP 8 | 1.8 (1.7) | 6.9 (1.7) | | CLS 8 | 0.4 (1.5) | 2.6 (1.5) | | CLS 8-9 | 0.1 (1.3) | -1.1 (1.2) | |
| CP 9 | 1.6 (1.3) | 5.8 (1.4) | | CLS 9 | 0.3 (1.7) | 3.7 (1.5) | | CLS 9-10 | -3.7 (1.7) | -7.4 (3.1) | |
| CP 10 | 1.5 (0.8) | 4.4 (1.2) | | CLS 10 | 4.0 (2.2) | 11.1 (3.0) | | ***AVG*** | ***0.1 (2.3)*** | ***3.0 (3.6)*** | |
| CP 11 | 0.0 (0.0) | 0.0 (0.0) | | ***AVG*** | ***0.0 (2.4)*** | ***-0.1 (6.7)*** | | | | | |
| ***AVG*** | ***0.8 (1.3)*** | ***6.0 (3.2)*** | | | | | | | | | |
| MDA Deviation | | | 0.012* | MDA Slope | | | 0.261 | MDA Bowing | | | 0.244 |
| CP 1 | 0.0 (0.0) | 0.0 (0.0) | | CLS 1 | 6.4 (3.1) | 21.4 (3.4) | | CLS 1-2 | -0.2 (3.4) | 13.3 (3.5) | |
| CP 2 | -2.4 (1.4) | 8.7 (1.6) | | CLS 2 | 6.5 (2.3) | 8.1 (1.8) | | CLS 2-3 | -0.7 (3.7) | 6.4 (1.8) | |
| CP 3 | 0.1 (1.4) | 11.8 (1.9) | | CLS 3 | 7.2 (2.2) | 1.7 (1.1) | | CLS 3-4 | 1.6 (2.0) | -0.8 (2.1) | |
| CP 4 | 2.9 (1.3) | 12.2 (2.2) | | CLS 4 | 5.6 (1.6) | 2.5 (1.5) | | CLS 4-5 | 1.2 (1.4) | -0.8 (2.2) | |
| CP 5 | 5.1 (1.5) | 11.2 (2.1) | | CLS 5 | 4.4 (1.2) | 3.3 (1.6) | | CLS 5-6 | 3.2 (1.2) | -0.4 (1.8) | |
| CP 6 | 6.8 (1.7) | 10.1 (1.9) | | CLS 6 | 1.2 (0.8) | 3.7 (1.5) | | CLS 6-7 | -0.9 (1.1) | -0.6 (0.9) | |
| CP 7 | 7.1 (1.8) | 8.6 (1.7) | | CLS 7 | 2.0 (0.9) | 4.4 (1.3) | | CLS 7-8 | -2.2 (1.3) | 0.1 (1.3) | |
| CP 8 | 6.3 (1.6) | 6.9 (1.6) | | CLS 8 | 4.2 (1.4) | 4.2 (1.5) | | CLS 8-9 | -0.2 (1.2) | 0.7 (1.5) | |
| CP 9 | 4.7 (1.3) | 5.3 (1.3) | | CLS 9 | 4.5 (1.4) | 3.6 (1.6) | | CLS 9-10 | -3.2 (3.1) | -6.4 (2.7) | |
| CP 10 | 3.0 (1.1) | 3.9 (1.0) | | CLS 10 | 7.7 (2.9) | 10.0 (2.7) | | ***AVG*** | ***-0.1 (1.8)*** | ***1.3 (5.2)*** | |
| CP 11 | 0.0 (0.0) | 0.0 (0.0) | | ***AVG*** | ***5.0 (2.0)*** | ***6.3 (5.6)*** | | | | | |
| ***AVG*** | ***3.1 (3.1)*** | ***7.2 (4.2)*** | | | | | | | | | |

Data are presented as mean (SD); all unpaired $t$-test $p$-value; $^*p < 0.05$
